# Characterizing the molecular mechanisms underlying aluminium-induced disruptions in neuronal survival pathways

**DOI:** 10.1007/s11011-026-01953-2

**Published:** 2026-08-27

**Authors:** Kankipati Sravya, Arpitha Rao, P. Harshitha, Aluru Parithathvi, Herman Sunil Dsouza

**Affiliations:** 1 Manipal School of Life Sciences, Manipal Academy of Higher Education, Manipal, Karnataka 576104 India; 2 Department of Radiation Biology and Toxicology, Manipal School of Life Sciences, Manipal Academy of Higher Education, Manipal, Karnataka 576104 India

**Keywords:** Aluminium, Signalling pathways, Apoptosis, Necroptosis, Ferroptosis, Pyroptosis

## Abstract

Aluminium, an omnipresent environmental pollutant, has been recognized for its neurotoxic effects, particularly its ability to promote neuronal death. More research evidence links aluminium exposure to death through molecular mechanisms. This review delves into the mechanisms underlying aluminium-induced neurotoxicity, including programmed cell death pathways such as apoptosis, necroptosis, autophagy, ferroptosis, and pyroptosis. Aluminium exposure leads to disruption of oxidative homeostasis and induction of endoplasmic reticulum stress, mitochondrial dysfunction and DNA damage, which in turn trigger different cell death pathways. Substantial changes in key signalling pathways, such as the IL-1β/JNK, PI3K/Akt/mTOR, Nrf2/Ho-1, System Xc-/GPX4, and NLRP3 inflammasome activation pathways, were observed. Overall, these intertwined pathways highlight aluminium as a potent modulator of neuronal survival pathways, driving neurodegeneration through multifactorial mechanisms. A thorough understanding of these changes provides a fundamental explanation of the role of aluminium toxicity in the etiology of neurodegenerative diseases and provides a foundation for treatment.

**Graphical abstract:**

**Figure:**
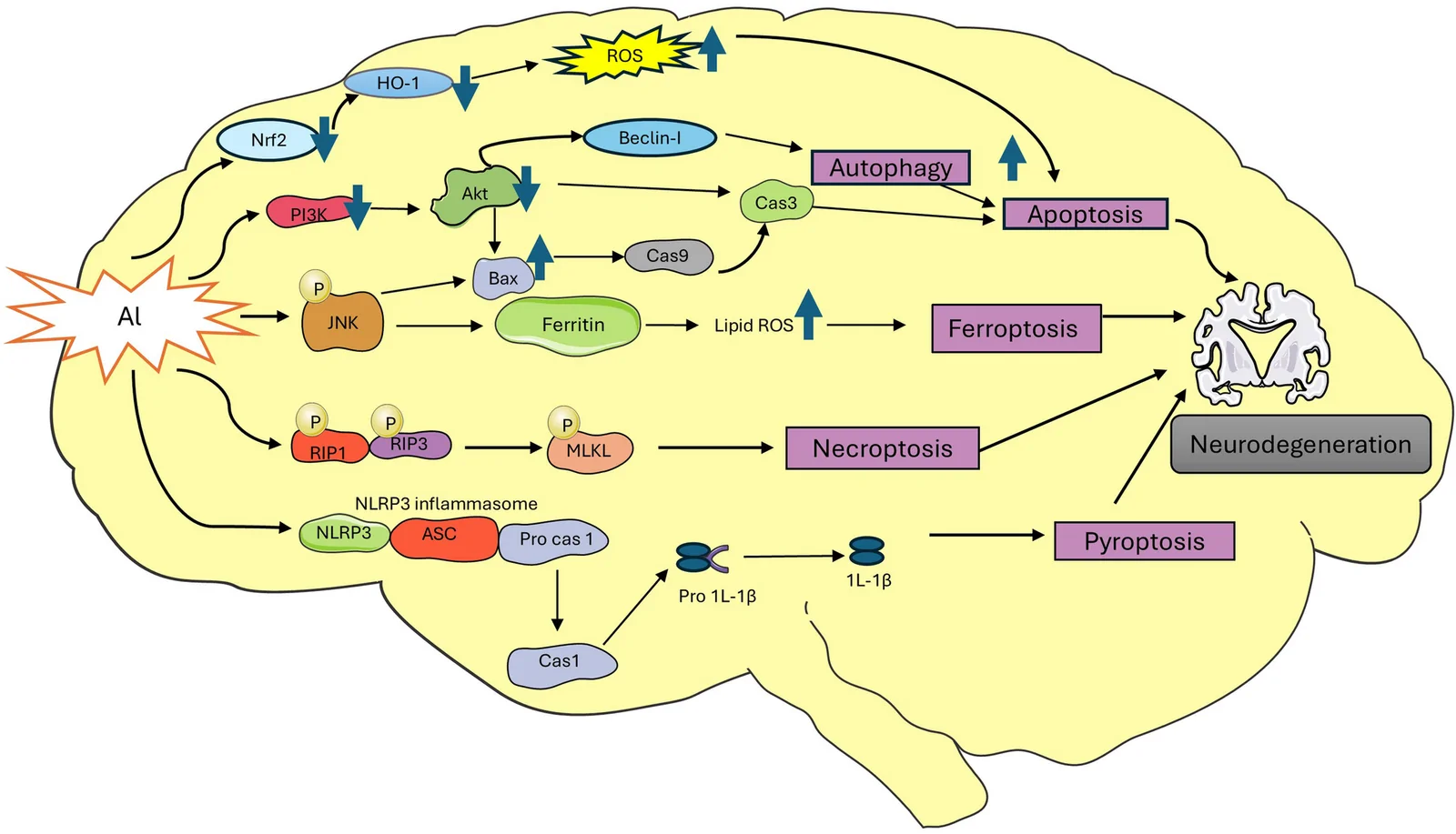

## Introduction

Aluminium is the predominant metal found in the Earth’s outermost layer. It is a white, lightweight, and highly reactive metal and is not readily available in its free state. It is an inessential metal that can cause toxicity when it accumulates in the body, and several studies suggest that aluminium exposure causes neurotoxicity. The cause of neurotoxicity from aluminium exposure is believed to be through oxidative stress, as aluminium forms a superoxide anion complex, which promotes reactive oxygen species (ROS) formation (He et al. 2023a; Kumar and Gill 2014). Aluminium accumulates in numerous parts of the brain, where it disrupts the levels of essential metals, resulting in molecular and structural damage, causing alterations in the brain (Dey and Singh 2022). The main route for aluminium uptake is thought to be across the blood–brain barrier (BBB). Studies have shown that aluminium increases the permeability of the BBB, leading to increased uptake, and it also has a longer retention time in the brain (Wang 2018). Neuronal death, a natural process, is a classic pathological feature of neurodegenerative diseases, and aluminium neurotoxicity leads to the death of various types of neurons (Zhang et al. 2020).

Aluminium and its compounds are used across various industries, such as food packaging, household goods, aviation, abrasive materials, and paint. The presence of aluminium in drinking water and food can be attributed to its use in processes such as packaging, food storage, and flocculation in water purification. (Krewski et al. 2007; Willhite et al. 2012). Aluminium hydroxide and aluminium phosphate are prevalent antacid drugs, antacids combined with aspirin, and vaccine adjuvants. Some aluminium formulations are used as food enhancers, and a few aluminium salts, such as aluminium chlorohydrate and aluminium zirconium, are used as antiperspirants (Flarend et al. 2001; Krewski et al. 2007). Aluminium enters the environment through anthropogenic and natural sources. Natural processes significantly exceed direct anthropogenic contributions to the environment. Direct anthropogenic emissions of aluminium compounds occur predominantly in the atmosphere and are linked to metallurgical processes such as smelting (Soni et al. 2001). Aluminium is introduced into the human body by ingestion, inhalation, and dermal contact and through adjuvanted vaccines via injection (Wang et al. 2020; Hao et al. 2023). Inhalation of aluminium is done majorly due to occupational exposure, and activities such as primary smelting for aluminium welding or foundry work are associated with increased uptake of aluminium through inhalation (Niu 2023). Furthermore, inhaled fine (< 10 μm diameter) aluminium particles are deposited in the lungs and affect the respiratory system. These particles can enter the alveolar region of the lungs, causing conditions such as aluminium pneumoconiosis or elemental uptake (Riihimäki and Aitio 2012). Aluminium enters our digestive system through the food and drinking water we consume. Aluminium oxides are used in food enhancers, preservatives, baking powders and emulsifiers. High aluminium intake can occur because of food item contamination during processing, cooking and storage. Leaching of aluminium can occur when acidic foods are prepared in aluminium pots, with concentrations reaching 50 mg/L (Crisponi et al. 2013). The use of aluminium based pharmaceutical products, such as antacids, phosphate binders, and aluminium contaminated dialysis fluids, can generally lead to aluminium toxicity (Reinke et al. 2003). Several studies suggest that aluminium exposure is linked to various systemic and neurodegenerative diseases. Aluminium\ toxicity is associated with osteoporosis, dyslipidemia, potroom asthma, nephrotic syndrome, renal glomerulonephritis, and neurodegenerative diseases such as Alzheimer’s disease, Parkinson’s disease, and multiple sclerosis (Inan-Eroglu and Ayaz 2018; Bondy and Campbell 2022; Jackson and Rout 2026). However, a direct link between aluminium exposure and these disorders remains to be explored. Previous studies have indicated that aluminium exposure in the brain can result in oxidative stress, mitochondrial dysregulation, and neuronal loss, which are characteristic features of neurodegenerative diseases (Skalny et al. 2021).

Currently, no effective treatment is available to reverse the neurological disorders caused by aluminium exposure. Antioxidants are suspected to be useful as adjuvant therapies because of their role in reducing oxidative stress, which is thought to play a central role in aluminium neurotoxicity (Fulgenzi et al. 2015). Certain phytochemicals, such as flavonoids and trace elements such as zinc and selenium, have been shown to exert protective effects against neuronal damage caused by aluminium in experimental studies because of their antioxidant and metal chelating properties (Zaitseva et al. 2025). Chelation therapy, especially with deferoxamine, is the main way to reduce the level of aluminium in the body and to prevent its accumulation in tissues (Jackson and Rout 2026).

**Fig. 1:**
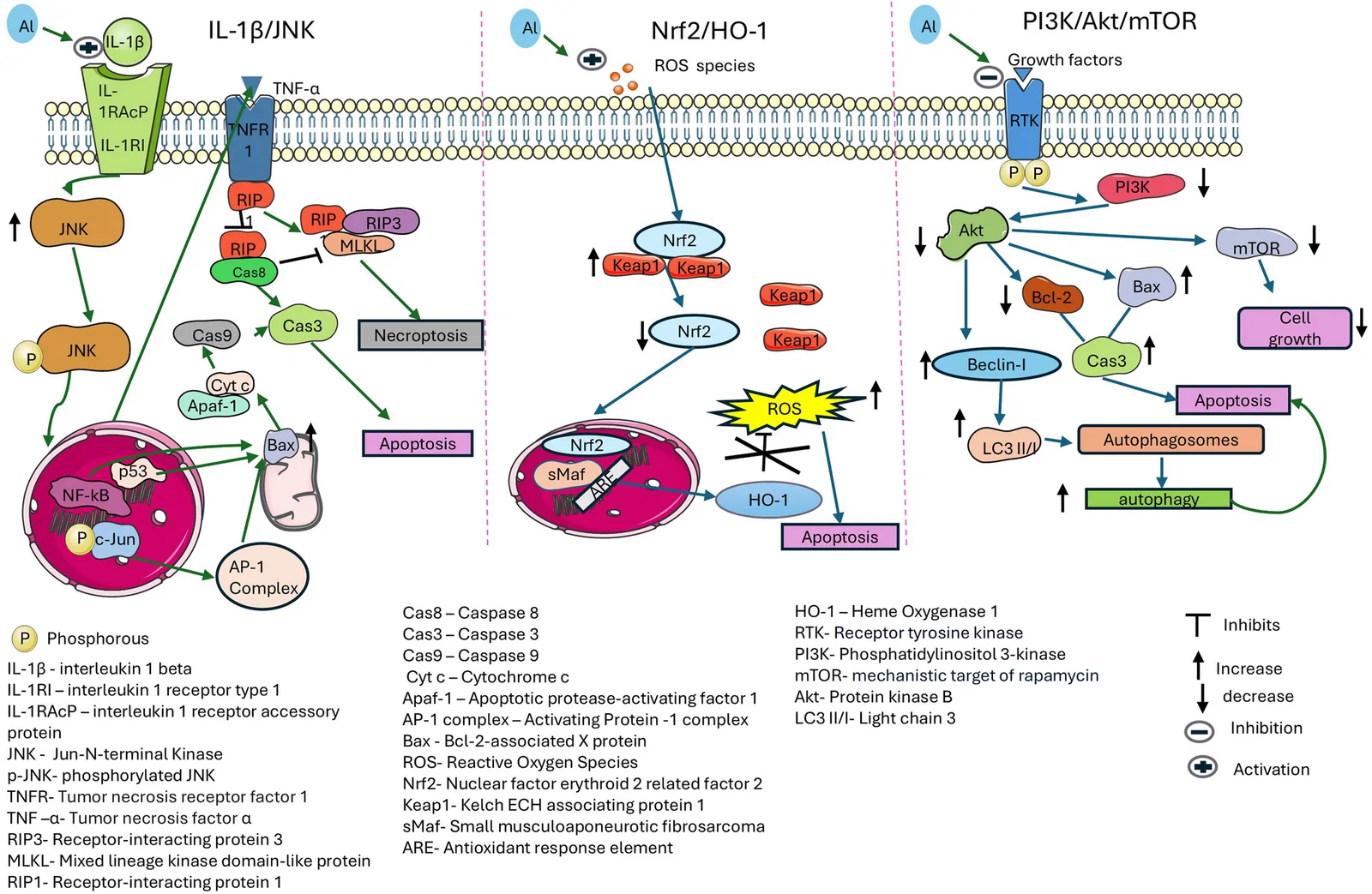
Schematic representation of aluminium-induced oxidative stress and cell death pathways. Aluminum exposure activates oxidative stress via increased ROS generation, disrupting Nrf2/ARE signalling and triggering downstream effects, including apoptosis, necroptosis, and autophagy. Key mediators such as TNF-α, RIP1/RIP3, MLKL, and caspases drive necroptosis and apoptosis, whereas alterations in the PI3K/Akt/mTOR and Beclin-1 pathways regulate autophagy. Dysregulation of Nrf2/HO-1 and JNK signalling further contributes to neuronal injury and inflammation

This review aims to study the role of aluminium in various types of neuronal death pathways (Fig. 1).

## Neuronal death and its pathways

Neuronal death is a natural process that occurs in the nervous system during development or is induced through various stimuli. The distinct types of neuronal death might act as factors in the mechanisms of neurological diseases. Multiple forms of neuronal death, such as apoptosis, necrosis, pyroptosis, parthanatos, ferroptosis, oncosis, lysosomal cell death, autophagy, phagocytosis and mitophagy, occur (Fricker et al. 2018).

### Apoptosis

Apoptosis, also known as programmed cell death, facilitates the removal of damaged or unused cells (Griffioen et al. 2004). It is easily distinguished from other cells because of its ability to cause cell shrinkage, damage to DNA, membrane blebbing, nuclear condensation, chromatin condensation, caspase activation, apoptotic body formation and DNA fragmentation. A crucial aspect of apoptosis is cell shrinkage (Bortner and Cidlowski 2003; Griffioen et al. 2004; Doonan and Cotter 2008). Changes in the mitochondrial membrane caused by apoptosis trigger the release of mitochondrial proteins and caspase-dependent activation, ultimately resulting in apoptotic death (Krysko et al. 2008). 

**Fig. 2:**
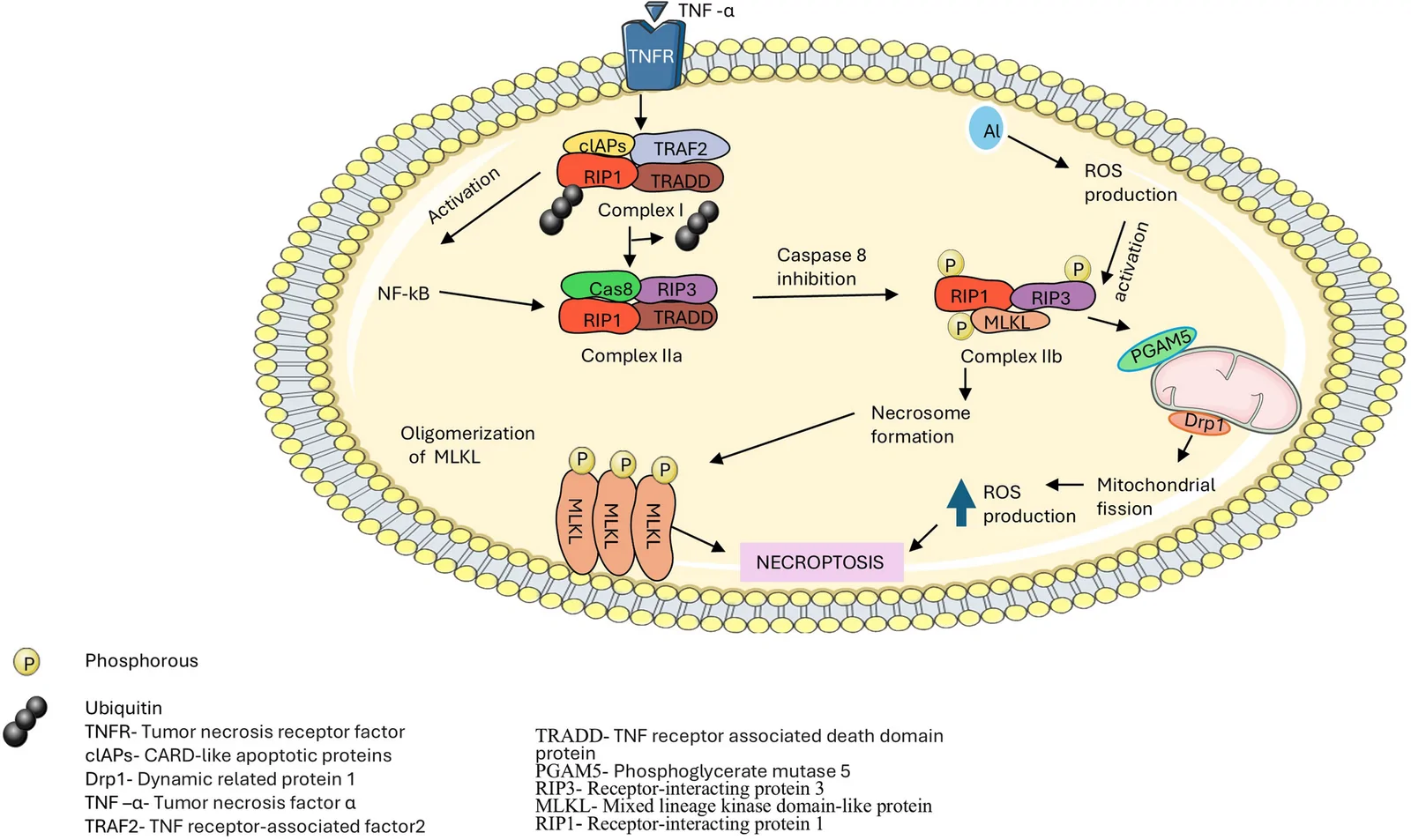
Schematic of the TNF-α–induced necroptosis signalling pathway. Inhibition of caspase-8 promotes necrosome formation via the RIP1-RIP3 interaction, leading to MLKL phosphorylation and oligomerization. Activated MLKL disrupts membranes, inducing necroptosis. The pathway involves TNFR, TRAF2, TRADD, and clAPs, with mitochondrial fission and ROS production mediated by PGAM5 and Drp1

As depicted in Fig. 2, aluminium induces autophagy and cell death through multiple converging pathways.

#### JNK pathway

The protein subfamily c-Jun N-terminal kinase (JNK) is a member of the mitogen-activated protein kinase (MAPK) superfamily and is involved in the modulation of cell proliferation, division and apoptosis. Apoptosis is regulated by many intrinsic signalling pathways, one of which is the JNK pathway. There are 3 isoforms of JNK, of which JNK 1 and JNK2 are produced everywhere, whereas JNK3 is produced predominantly in neuronal and cardiac tissues (Liu and Lin 2005). The type of cell and stimuli involved in activation determine the fate of activated JNKs, leading to either apoptosis or cell proliferation. Numerous studies have provided evidence that all three isoforms are involved in stimulating apoptotic signalling. JNK stimulates the activation of pro-apoptotic genes such as *Bax* and suppresses the expression of anti-apoptotic genes such as *Bcl2*. It also promotes apoptosis by inducing the release of cytochrome c from the mitochondrial membrane and phosphorylating and regulating the activities of various pro- and anti apoptotic proteins. JNK participates in the apoptotic pathway and ensures efficient execution of apoptosis (Dhanasekaran and Reddy 2008).

#### *Nrf2* pathway

Nuclear factor erythroid 2 related factor 2 (*Nrf2*) serves as a transcription factor responsible for modulating cellular redox homeostasis in mammals. It is also responsible for regulating many genes present in the antioxidant response element (ARE) region. Cysteine-rich Kelch ECH-associated protein 1 (Keap1) functions as a suppressor protein that attaches to *Nrf2* and facilitates its breakdown by the ubiquitin proteasome pathway (Loboda et al. 2016). *Nrf2* induces phase-II antioxidant proteins that are responsible for clearing ROS. The association between *Nrf2* and Keap1 is disrupted during oxidant exposure. Inactivation of this pathway increases ROS levels, leading to neuroinflammation, immune dysregulation, demyelination, axonal degeneration, spinal cord injury, mitochondrial dysfunction, apoptosis, neurotransmitter imbalance, and increased inflammatory response and aging (Wang et al. 2019; Upadhayay and Mehan 2021).

#### Phosphatidylinositol 3-kinase/protein kinase B (PI3K/Akt) pathway

The PI3K/Akt signalling pathway is a crucial intracellular signal transduction cascade that is essential for the modulation of apoptosis and cell longevity. The serine/threonine-specific protein kinase Akt, otherwise known as protein kinase B (PKB), serves as a crucial effector in PI3K signalling. It promotes cell survival either through the activation of apoptosis cascade regulators or through the negative regulation of proapoptotic genes. Inhibiting this pathway can induce apoptosis. Activation of PI3K occurs in several signalling pathways mediated by growth factor receptors, leading to the activation of Akt. Akt activity controls PI3K-dependent cell viability (Meng et al. 2017).

### Necrosis

Necrosis is an uncontrolled type of cell death activated by pathological processes. It is characterized by swelling of cell organelles, breakdown of the cell membrane and ultimately cell lysis, resulting in cell damage (Khalid and Azimpouran 2024). Necroptosis is caused by the activation of tumor necrosis factor-α (TNF-α), an executor of the inflammatory response. TNF-α is responsible for triggering receptor-interacting protein kinase 1 (RIP1) and receptor-interacting protein kinase 3 (RIP3), as well as mixed lineage kinase domain-like (MLKL) protein (Fig. 2) (Zhang et al. 2024a). TNF-α is a death receptor ligand that binds to its receptor tumor necrosis factor receptor (TNFR). This binding results in the recruitment of other proteins, such as TNF receptor-associated death domain protein (TRADD), RIP1, CARD-like apoptotic proteins (CLAPs) and TNF receptor-associated factor 2 (TRAF2), and leads to the formation of complex I. Complex I initiates NF-kB, and the activated NF-kB pathway or deubiquitination of RIP1 results in the formation of complex IIa, which consists of RIP1, RIP3, TRADD and caspase 8. In general, caspase 8 leads to the activation of the apoptotic pathway, but the inhibition of caspase 8 leads to complex IIb formation (Zhou et al. 2022). RIP1 detaches from complex IIa and binds to RIP3, which undergoes autophosphorylation and recruits MLKL. MLKL proteins are phosphorylated and oligomerize on the membrane and form pores, causing cell rupture and leading to necroptosis (Hu et al. 2020).

### Ferroptosis

**Fig. 3:**
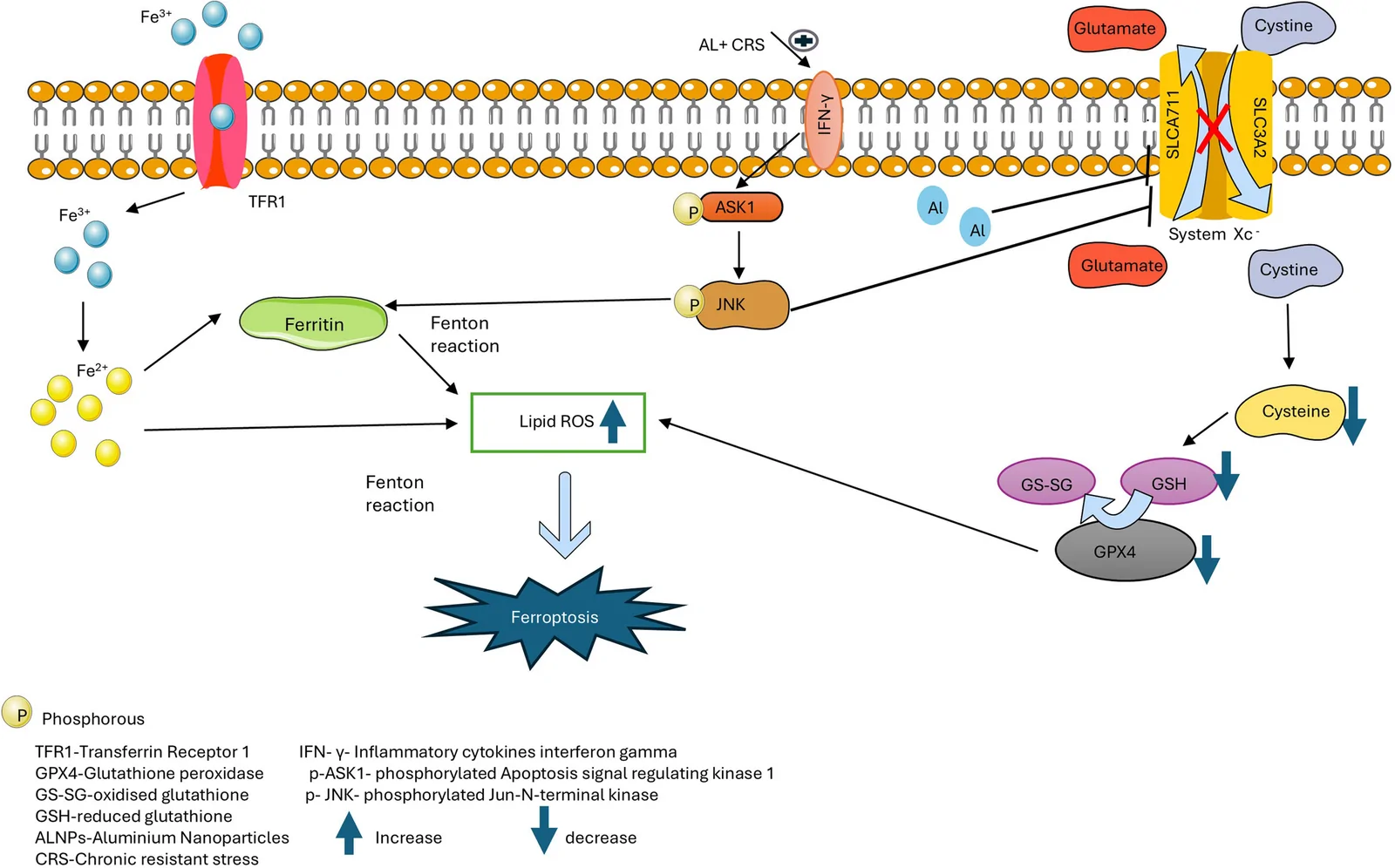
Mechanism of ferroptosis in neuronal cells under aluminium and chronic stress Aluminum levels and chronic resistance stress (CRS) increase lipid ROS generation through disrupted iron homeostasis (through TFR1) and Fenton reactions. Impaired System Xc− function reduces cystine uptake, depleting GSH and inhibiting GPX4, leading to ferroptosis This pathway is further amplified by IFN-γ–mediated activation of ASK1 and JNK signalling

Ferroptosis is a mode of programmed cell death activated by iron reactive oxygen species, which rely on iron or lack the ability of cells to scavenge glutathione peroxidase 4 (GPX4) (Dong et al. 2024). It is morphologically vastly different from other modes of cell death. This is indicated by the disruption of the mitochondrial membrane and the disappearance of mitochondrial cristae (Tang et al. 2018; Zhang et al. 2021) (Fig. 3). Ferroptosis is regulated by iron, lipid and amino acid metabolism, which are controlled by Systems Xc^−^ and p53. System Xc^−^ is an amino acid antiporter involved in the import of cystine and the export of glutamate through the plasma membrane. The import of cystine is converted into cysteine and is responsible for the production of reduced glutathione (GSH). The Fe^3+^ ions present in our body can be coupled with transferrin, leading to the uptake of Fe^3+^ ions through transferrin receptor 1 (TFR1). Six-transmembrane epithelial antigen of prostate 3 (STEAP3), located within the receptor, reduces Fe^3+^ ions to Fe^2+^ ions. The released Fe^2+^ is either released in the cytoplasm or stored in ferritin. The free Fe^+ 2^ ions and ions released from ferritin by ferroportin undergo the Fenton reaction and produce ROS such as hydroxyl and superoxide, which cause ferroptosis (Chen et al. 2022; Wang et al. 2024).

### Pyroptosis

**Fig. 4:**
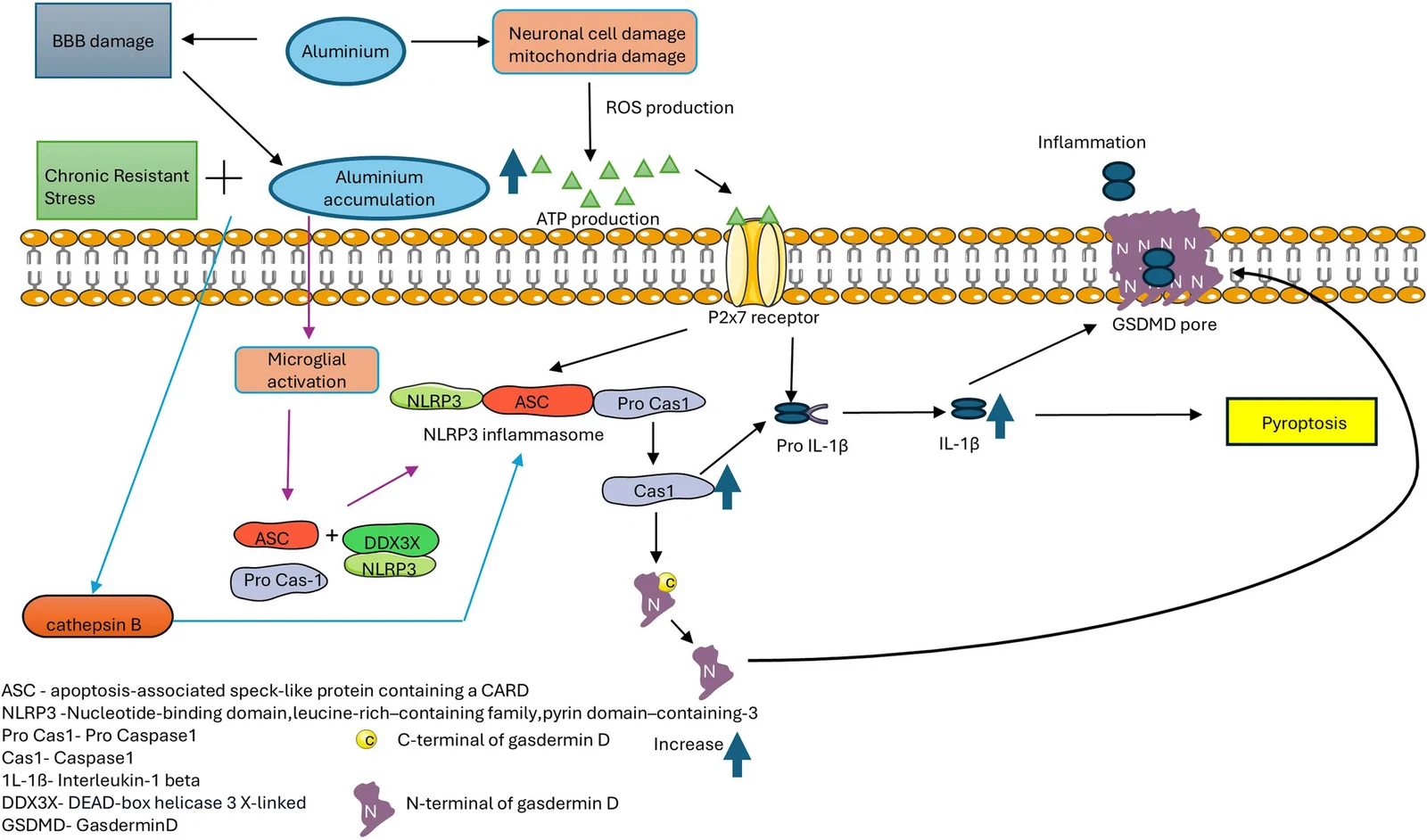
NLRP3 inflammasome-mediated pyroptosis in neuronal cells. Aluminum-induced stress triggers mitochondrial damage, ROS production, and cathepsin B release, activating the NLRP3 inflammasome. This leads to caspase-1 activation, IL-1β maturation, and gasdermin D cleavage. The N-terminus of gasdermin D forms membrane pores, inducing pyroptosis Associated effects include microglial activation, BBB disruption, and neuronal damage

The term pyroptosis was first articulated in 2001, and the term “pyroptosis” refers to inflammation. Pyroptosis is characterized by cell lysis (Hao et al. 2021; Dai et al. 2023). Inflammation is caused by the activation of the inflammasome through the capase-1 and canonical nucleotide-binding domain, leucine-rich–containing family, pyrin domain–containing protein 3 (NLRP3) inflammasome pathways, resulting in the activation of gasdermin D (GSDMD), which functions as the executor of pyroptosis (Hao et al. 2023; Zhang et al. 2024b) (Fig. 4). Cleavage of GSDMD results in the development of pores within the cell membrane, resulting in the pyroptotic death of cells (Wongpun et al. 2023). Toll-like receptor (TLR4), IL-1, caspases, TNF-α, lipopolysaccharide (LPS), and IL-1β are responsible for upregulating NLRP3 inflammation in different ways, such as through canonical, non-canonical and alternate pathways (Que et al. 2024). DEAD-box helicase 3X-linked (DDX3X) is a protein encoded by genes located on the X chromosome and is a member of the DEAD box helicase family. DDX3X is a common component of stress granules and can activate NLRP3 (Samir et al. 2019). P2X receptors are ligand-gated cation channels, and purinergic P2X receptor 7 (P2 × 7) is linked to chronic inflammatory neurological disorders (Zhu et al. 2023).

## Methodology

A systematic search of the literature was performed to evaluate the alterations in pathways associated with neuronal death induced by aluminium.

### Database search

An extensive search strategy was used to explore the role of aluminium in inducing neuronal death. Three major scientific databases, PubMed, Science Direct, and Scopus, were used for this purpose. The search encompasses articles published between 2012 and 2024.

### Keywords and search terms

Various keyword combinations were applied to ensure a focused and comprehensive search. The main terms included “aluminium” combined with “cell death,” “apoptosis”, “necrosis”, “pyroptosis”, “ferroptosis”, “autophagy”, and “neurodegeneration”.

Boolean operators (AND, OR) were systematically used to connect and refine these terms, allowing for the precise retrieval of relevant studies while maintaining comprehensive coverage of the topic.

Following the initial retrieval of 17,477 articles, predefined selection criteria were applied to identify the most relevant studies.

### Inclusion and exclusion criteria

Eligible studies included human studies, in vitro experiments, and in vivo animal models, particularly studies involving rodents such as rats, mice and zebrafish. Studies investigating the combined effects of aluminium with other metals or chemical agents were excluded to ensure that the observed alterations in neuronal death pathways could be attributed specifically to aluminium exposure. Duplicate records identified during the database search were removed.

**Fig. 5:**
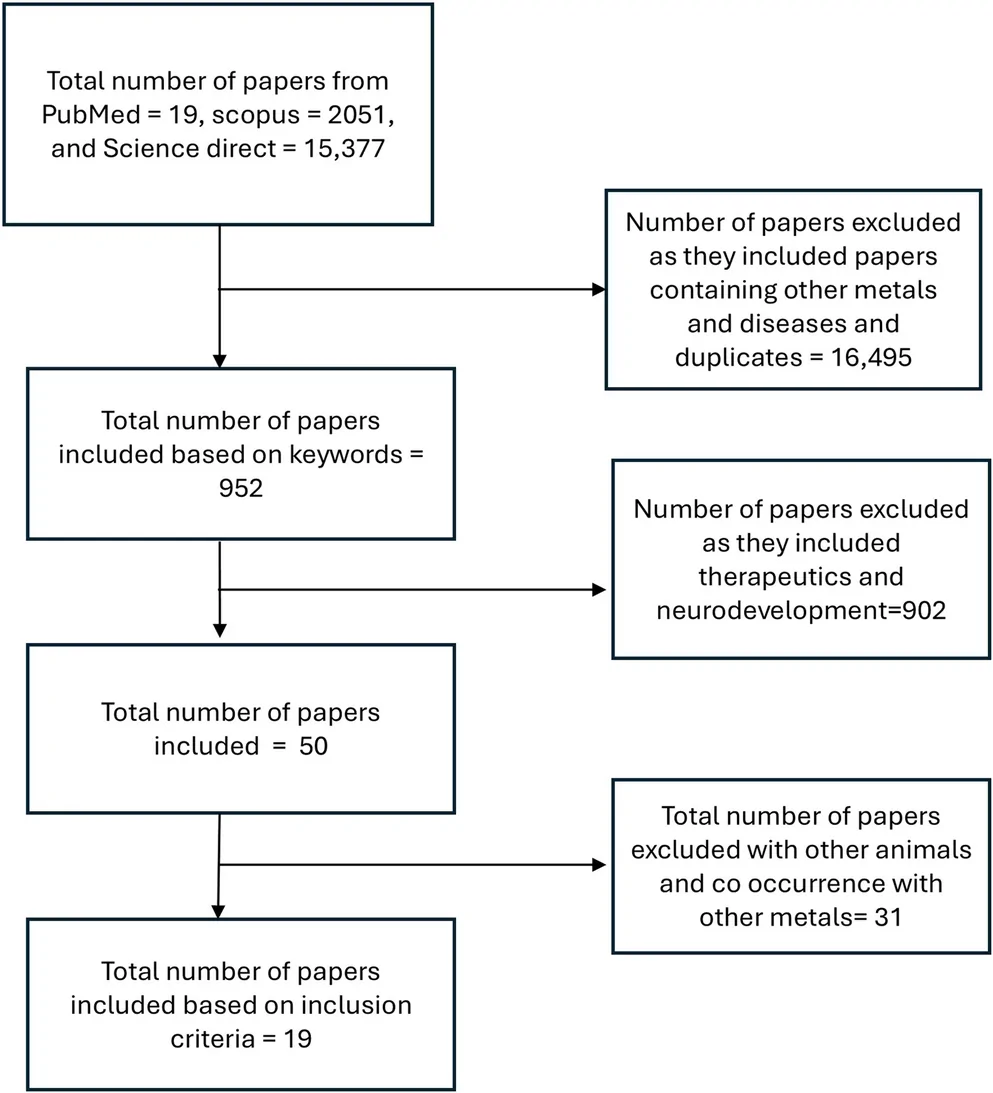
The systemic selection of the papers was sourced from various databases and finalised using PRISMA guidelines

Nineteen papers were shortlisted on the basis of the inclusion and exclusion criteria using Rayyan, as illustrated in Fig. 5 and summarised in Table 1. The papers were selected individually by the first authors to avoid bias.

**Table 1:** Compilation of selected papers on aluminium induced neuronal death, highlighting study type, exposure dose, type of study and affected pathways

Aluminium dosage	Type of study	Pathway	Result	Reference
450 mg/kg < 150 mg/kg	In vivo (Wistar rats)	IL-1β/JNK	• Increased levels of IL-1β/JNK signalling pathway proteins. • High dose induced necroptosis. • Low dose induced apoptosis.	(Zhang et al. 2020)
100µM, 200µM, 400µM, 800µM,	In vitro (PC 12 cells)	*Nrf2*/HO-1	• Reduced Nrf2 levels and HO-1 levels. • Elevated ROS and activated apoptosis. • 400µM showed significant results.	(He et al. 2023b)
0 µg/L, 20 µg/L, 100 µg/L, 500 µg/L	In vivo (Zebrafish)	PI3K/Akt/mTOR	• Dose dependent reduction in PI3K, Akt, and mTOR1 genes were observed with increase in concentrations.	(Shang et al. 2020)
10µmol/kg 20µmol/kg, 40µmol/kg	In vivo (Sprague-Dawley rats)	PI3K/Akt/mTOR	• Reduction in PI3K, Akt, and mTOR protein levels.	(Li et al. 2020)
200µM, 400µM, 800µM, 1600µM	In vitro	Beclin 1-dependent autophagy	• Elevated levels of Beclin 1. • Activation of autophagy followed by apoptosis. • Activated autophagy at 400µM and apoptosis at 1600µM.	(Zeng et al. 2012)
No dose mentioned	In vitro (L929 cells)	TNFR1-RIP1/RIP3 pathway	• Increased expressions of TNFR1, RIP1, RIP3, and MLKL. • Activation of necroptosis.	(Hu et al. 2020)
100µM 200µM 400µM	In vitro (PC 12 cells)	RIP3-PGAM5-Drp1 pathway	• Elevated ROS levels. • Increased expressions of RIP1, RIP3, MLKL, PGAM5, and Drp1. • PGAM5 activates Drp1. • Activation of necroptosis	(Zhang et al. 2024a)
200µM	In vitro (PC12 cells)	System Xc^−^ pathway	• Disruption of System Xc. • Downregulation of antioxidants. • Increase in lipid peroxidation. • Activation of ferroptosis.	(Cheng et al. 2021)
50 mg/kg	In vivo (Male Wistar rats)	IFN-γ/ASK/JNK pathway	• Upregulation of IFN-γ. • Activation of INF-γ/ASK/JNK pathway. • Activation of ferroptosis.	(Zhang et al. 2021)
2mM 303 mg/kg	In vitro (BV2 microglial cells) In vivo (Kunming mice, C57BL/6 mice)	DDX3X-NLRP3 pathway	• Co localization of DDX3X and NLRP3. • Activation of pyroptosis.	(Hao et al. 2021, 2023)
2mM 303 mg/kg	In vitro (BV2 microglia cells, HT22 cells) In vivo (Kunming mice)	P2 × 7-NLRP3 pathway	• Upregulation of IL-1β, and ATP. • Activation of P2 × 7. • Production of inflammatory cytokines.	(Zhu et al. 2023)
250µM 50 mg/kg	In vitro (HAPI cells) In vivo (Male Wistar rats)	Cathepsin B/NLRP3 signaling pathway	• Activation of cathepsin B. • Production of inflammatory cytokines.	(Zhang et al. 2022)
100µM, 200µM	In vitro (SH-SY5Y cells)	Prolonged activation of UPR	• Upregulation of ERS. • Activation of apoptosis through UPR.	(Rizvi et al. 2016)
10.73 mg/kg 107.33 mg/kg 1073.33 mg/kg	In vivo (SD rats)	P53 Phosphorylation	• Increased expression of p53. • High dosage group showed higher expression of p53.	(Pan et al. 2015)
4mmol/L	In vitro (SH-SY5Y cells)	ERK and p38 MAPK	• ERK activates apoptosis. • ERK and MAPK together activate necroptosis.	(Jia et al. 2014)
20µmol/kg, 40µmol/kg, 80µmol/kg	In vivo (C57BL/6 mouse)	RIP/RIP3/MLKL pathway	• Medium and high dose groups showed increased expression of RIP3, HSP90 and MLKL. • Activation of necrosis.	(Zhang et al. 2023)
50 mg/kg, 150 mg/kg, 450 mg/kg	In vivo (Sprague Dawley rats)	Neuroinflammation	• Inflammation and loss of dendritic spines. • Activation of apoptosis	(Cao et al. 2016)
50 mg/kg, 150 mg/kg, 450 mg/kg	In vivo (Male Wistar rats)	Hippocampal Inflammatory lesions	• Induction of inflammatory lesions.	(Zhang et al. 2018)

## Role of aluminium in neuronal death pathways

Aluminium toxicity leads to alterations in pathways, ultimately leading to cell death. Human exposure to aluminium can occur through various sources, ranging from occupational conditions to the presence of aluminium in daily use and consumption products.

### Apoptosis

A study by Zhang et al. focused on the involvement of aluminium in apoptosis and necroptosis. Interleukin-1β (IL-1β) binds to specific receptor and receptor accessory proteins, such as IL-1RI and IL-1RAcP, to trigger a downstream signalling cascade, which leads to JNK activation. Activated JNK can modulate the expression of Bcl-2-associated X (*Bax)* and B-cell lymphoma 2 (*Bcl2)* and activate a caspase cascade to regulate apoptosis. Chronic activation of JNK affects the expression of TNF-α. TNF-α binds to TNFR1, a chief mediator of the inflammatory pathway, and under conditions favourable for apoptosis, it engages RIP1 and triggers caspase-8, which suppresses the upregulation of RIP1 and RIP3 and triggers the caspase cascade, leading to apoptosis. AlCl_3_ was found to cause necroptosis in hippocampal neural cells, as it increased the protein and mRNA expression levels of TNF-α, TNFR1, RIP1, RIP3, and MLKL and decreased the protein level of caspase-8. Aluminum exposure led to increased levels of the core proteins of the IL-1β/JNK signalling pathway. Compared with those in the controls, the protein expression levels of IL-1β, IL-1RI and IL-1RAcP were considerably elevated in the aluminium-treated groups (Zhang et al. 2020). Aluminium inhibits Nrf2/HO-1 signalling, which results in apoptosis. At specific concentrations, Nrf2 is known to decrease the apoptosis of pheochromocytoma (PC12) cells induced by H_2_O_2_. It is also a strong modulator of the cellular oxidative stress response. In hippocampal neurons, β-amyloid-induced mitochondrial dysregulation and oxidative stress were reduced by stimulation of the Nrf2/HO-1 signalling pathway. As the concentration of aluminium increased, the relative levels of Nrf2 and HO-1 protein expression gradually decreased. It is unclear exactly how the pathway affects the *Bax* and *Bcl2* proteins, but it could be due to HO-1 and *Bax* cross talk or, as another theory suggests, the decrease in the antioxidant capacity of HO-1, which results in the accumulation of ROS and the activation of the JNK pathway, in turn triggering apoptosis (He et al. 2023b). In a study performed by Shang et al. involving workers with occupational aluminium exposure, cognitive function was assessed through the Mini-Mental State Examination (MMSE) and Clock-Drawing Test (CDT), and serum aluminium levels and PI3K/Akt/mTOR-associated gene activation were measured. The results indicated downregulation of the PI3K/Akt/mTOR pathway. Compared with control workers, aluminium-exposed workers presented significantly higher serum aluminium levels and lower expression of the Phosphoinositide 3-kinase (PI3K), Akt and mechanistic target of rapamycin (mTOR1) genes. These findings suggest that elevated aluminium concentrations in the brain are related to inhibition of signalling, which can then lead to neuronal loss (Shang et al. 2020). Li et al. reported that the levels of PI3K, Akt, and mTOR in rats were considerably lower in the aluminium treated group than in the control group. These findings indicate that exposure to aluminium substantially affects the proteins of the PI3K/Akt/mTOR signalling pathway, which are related to the regulation of the neuronal synaptic plasticity process. p-p85 Tyr467 for PI3K, p-Akt Thr308 and p-Akt Ser473 for AKT and p-mTOR Ser2448 for mTOR are specific phosphorylation sites for these proteins. Western blotting revealed that the phosphorylation of these proteins at these sites was highly elevated in the aluminium treated group. These findings indicate that despite the reduction in the total protein concentration of PI3K, Akt, and mTOR, the phosphorylation levels of these three proteins increased in a dose-dependent manner. The results of the current research suggest that the PI3K/Akt/mTOR pathway may be linked to the damage caused by aluminium on synaptic plasticity, leading to cell death (Li et al. 2020). Rat astrocytes treated with aluminium presented elevated levels of autophagy and apoptotic proteins. A group of researchers conducted a study in a rat model in which aluminium was induced into astrocytes at a low dose of 400 µM, leading to Beclin1 activation, which is a class III PI3K protein that causes an increase in LC3II expression. It is at this concentration of aluminium that apoptotic proteins were not expressed. With a further increase in the concentration of aluminium to approximately 800 µM, the Beclin1 level increased and led to the formation of autophagosomes, in turn leading to autophagy and increasing the amount of apoptosis associated with proteins, such as cleaved caspase-3 and cleaved PARP, which are late apoptotic proteins. At a very high concentration 1600 µM, the levels of cleaved caspase-3 and cleaved PARP were elevated, indicating that the autophagy caused by aluminium was subsequently succeeded by apoptosis. These results indicate that extreme autophagy may cause apoptosis through Beclin1 mediated autophagy signalling (Zeng et al. 2012). Aluminium can induce oxidative stress and ERS, which release ERS proteins such as PERK and ELF2α, leading to the induction of the unfolded protein response (UPR). Prolonged activation of the UPR results in the release of apoptotic proteins such as caspase 3 and caspase 9, leading to apoptosis. It also activates the NF-kB and NLRP3 pathways, resulting in inflammation (Rizvi et al. 2016). Another study reported an increase in the protein expression of p53 and Pp53-ser15 due to the presence of aluminium in the cortex, which can induce neuronal apoptosis in rats (Pan et al. 2015). The deposition of AlCl3 in the hippocampus results in neuroinflammation and the release of inflammatory factors such as IL-1β, TNF-α, and IL-6, which would influence the performance of the hippocampus and result in the loss of dendritic spines and apoptosis (Cao et al. 2016). AlCl3 caused hippocampal neuron loss and hippocampal inflammatory lesions (HILs), and these HILs were linked to the induction of the IL-1 signalling pathway (Zhang et al. 2018).

These studies suggest that aluminium induced apoptosis is related to the failure of neuronal survival mechanisms. Zhang et al. emphasised IL-1β/JNKmediated inflammatory apoptosis, whereas Shang et al. and Li et al. linked aluminium exposure with PI3K/Akt/mTOR disruption, and He et al. highlighted the suppression of the Nrf2/HO-1 antioxidant pathway. Furthermore, evidence from Zeng et al. suggests that aluminium-induced neuronal responses may be concentration dependent, with lower concentrations favouring autophagy and higher concentrations progressively promoting apoptotic cell death. Together, these findings indicate that apoptosis is driven by oxidative stress, inflammation, mitochondrial dysfunction and impaired survival signalling in a concentration-dependent manner.

### Necrosis

The PC12 cells were subjected to Al(mal)_3,_ and shortening of the PC12 cells occurred, accompanied by disruption of the cell membrane. Aluminium was found to promote TNFR1-RIP1/RIP3 signalling pathway-induced necroptosis through targeting of PC12 cells with Al(mal)_3_, indicating that aluminium can increase the expression levels of TNFR1, RIP1, RIP3 and MLKL (Hu et al. 2020). Aluminium triggers necroptosis through the RIP3-PGAM5-Drp1 pathway. Phosphoglycerate mutase 5 (PGAM5) is an enzyme that activates mitochondrial fission and controls metabolic processes involving cell death, mitosis and aging. Dynamic related protein 1 (Drp1) is an essential protein that can be found in the cytosol and is linked to mitochondrial fission and division. Zhang et al. reported that exposure to aluminium increased the formation of ROS, leading to increases in the concentrations of RIP1, RIP3, MLKL, PGAM5, and Drp1 and the distortion of the mitochondrial membrane. RIP3 is important for necroptosis. When activated, RIP3 recruits PGAM5, which in turn activates Drp1, leading to mitochondrial cleavage, an execution step in necroptosis (Zhang et al. 2024b). Jia et al. experimented on the SH-SY5Y cell line and reported that the ERK and p38 MAPK signalling pathways are involved in the induction of necroptosis by aluminium (Jia et al. 2014). The role of the Hsp90 protein in the necroptosis triggered by aluminium through RIP/RIP3/MLKL was also reported by Zhang et al. (Zhang et al. 2023).

According to necroptosis investigations, aluminium promotes inflammation and death pathways linked to mitochondrial injury. The TNFR1-RIP1/RIP3/MLKL axis is mostly supported by the findings of Zhou et al., while Zhang et al. emphasised the RIP3-PGAM5-Drp1 route as a mechanism linked to mitochondrial fission. These results imply that both death receptor signalling and mitochondrial fragmentation may be responsible for aluminum-induced necroptosis. The relationships among RIP3 activation, ROS production, MLKL phosphorylation, and Drp1-mediated mitochondrial disruption suggest that oxidative stress and mitochondrial failure, which are also essential for ferroptosis and apoptosis, overlap with necroptosis.

### Ferroptosis

In an in vitro experiment by Cheng et al. PC12 cells from rats responded to different concentrations (200 µM and 400 µM) of Al(mal)_3_. Erastin is an inducer of ferroptosis, and the cells were induced with Al(mal)_3_ along with erastin to observe the ferroptotic pathway resulting from Al(mal)_3_. The survival of the cells was assessed, and a drastic reduction in the viability of the cells exposed to Al(mal)_3_ and the combination of Al(mal)_3_ and erastin were observed. These two groups also showed changes in mitochondria, such as disruption of the membrane and a reduction in the number of mitochondrial ridges. Damaged mitochondria are more efficient at producing ROS and triggering ferroptosis. A decrease in the protein level of SLC7A11, a subunit of System Xc^−,^ was observed upon exposure to Al(mal)_3_; in addition, activated oxidative damage due to ROS accumulation disrupted the system Xc^−^ amino antiporter. The disruption of System Xc^−^ further inhibited the uptake of cystine, reduced the production of GSH and inhibited the function of GPX4, resulting in the accumulation of toxic lipid peroxides and accelerating the process of ferroptosis (Cheng et al. 2021). A study by Zhang et al. concluded that aluminium nanoparticles (ALNPs) and chronic resistance stress (CRS) together contribute to ferroptosis through the IFN-γ/ASK/JNK pathway (Inflammatory cytokines interferon gamma/apoptosis signal regulating kinase 1/Jun-N-terminal kinase). The presence of ALNPs in the hippocampal region leads to the production of the inflammatory cytokines IL-1β, IL-16, TNF-α and IFN-γ, and synergistic exposure with CRS in the hippocampal region increases the generation of IFN-γ. The release of IFN-γ activates ASK1 via phosphorylation, leading to the activation of JNK. Activated JNK can cause ferritin degradation, leading to iron accumulation and further ferroptosis. Combined exposure of ALNPs and CRS causes iron metabolism dysregulation and decreases the protein expression of SLC7A11, causing a reduction in the levels of antioxidants, which results in increased ROS and lipid peroxidation levels and leads to hippocampal ferroptosis via the IFN-γ/ASK/JNK pathway (Zhang et al. 2021).

Ferroptosis studies have revealed that aluminium induced ferroptosis is caused mainly by lipid peroxidation, inflammatory amplification, and antioxidant failure. Reduced SLC7A11 expression reduces cystine uptake, lowers GSH synthesis, and weakens GPX4-mediated lipid peroxide clearance, all of which disturb the system Xc−/GSH/GPX4 axis. On the other hand, Zhang et al. demonstrated that alumina nanoparticles and long-term stress trigger the IFN-γ/ASK1/JNK pathway, which connects inflammation to iron dysregulation and lipid ROS buildup. Ferroptosis thus serves as a link between mitochondrial damage, oxidative stress, inflammatory cytokine signalling, and compromised antioxidant defense.

### Pyroptosis

In an experiment by Hao et al., the induction of aluminium in BV-2 microglial cells and the cerebral cortex was conducted. The accumulation of aluminium in the brain results in neuroinflammation through the triggering of microglia by damaging the tight junction of the BBB. Microglial damage results in the release of proinflammatory factors and the activation of DDX3X-NLRP3 signalling. DDX3X colocalizes with NLRP3 to activate the NLRP3 complex, cleaves caspase-1, transforms proinflammatory substances into inflammatory substances and cleaves the C-terminus of GSDMD to form the N-terminus of GSDMD. The N-terminus creates pores on the membrane, releasing inflammatory molecules to induce pyroptotic death (Hao et al. 2021, 2023). The mechanism through which aluminium induces the P2 × 7-NLRP3 pathway was explained in a study by Zhu et al., in which aluminium was introduced into BV2 cells, hippocampal neuronal cell line (HT22) and the cerebral cortex of mice. Exposure to aluminium reduced cell viability and triggered microglia, which in turn triggered an inflammatory response of IL-1β and elevated the production of ATP, which activated the P2 × 7 receptor. Instigation of the P2 × 7 receptor results in inflammatory cytokine release and subsequent cell death. The NLRP3 complex is triggered by the P2 × 7 receptor and hypoxia inducible factor 1α (HIF-1α) to trigger an inflammatory response and neuronal damage (Zhu et al. 2023). Aluminium can cause pyroptosis through the cathepsin B/NLRP3 signalling pathway. CRS, along with ALNPs, causes cell death in microglia by activating cathepsin B. Cathepsin B is a signalling factor of NLRP3; upon activation of cathepsin B, the NLRP3 inflammasome is activated, and proinflammatory substances are converted to inflammatory substances, resulting in an inflammatory response. On the other hand, the cleaved N-terminus of GSDMD forms pores on the membrane, releasing inflammatory substances (Zhang et al. 2022).

Studies on pyroptosis have shown that NLRP3 inflammasome signalling and microglial activation play a major role in mediating aluminum-induced inflammatory cell death. Zhu et al. identified the P2 × 7-NLRP3 pathway, Zhang et al. reported cathepsin B/NLRP3 activation during combined exposure to alumina nanoparticles and chronic stress, and Hao et al. highlight DDX3X-NLRP3-mediated pyroptosis. Despite these studies concentrating on various upstream activators, they focused on GSDMD-mediated pore formation, caspase-1 cleavage, IL-1β maturation, and NLRP3 inflammasome activation. This implies that NLRP3 functions as a key inflammatory hub in pyroptosis induced by aluminium and may also enhance ferroptosis, necroptosis, and apoptosis via cytokine production and persistent neuroinflammation.

## Integration of aluminium induced neuronal death pathway

Aluminium-induced neurotoxicity is driven by interconnected molecular mechanisms rather than isolated cell death pathways. Oxidative stress appears to be a common upstream event, as aluminium promotes ROS generation, mitochondrial dysfunction, and impairment of antioxidant defence systems (Kumar and Gill 2014; He et al. 2023b). Suppression of the Nrf2/HO-1 pathway further enhances ROS accumulation, contributing to apoptosis through mitochondrial damage and caspase activation, while also promoting ferroptosis through disruption of the system Xc−/GSH/GPX4 axis and lipid peroxidation (He et al. 2023a; Cheng et al. 2021).

**Fig. 6:**
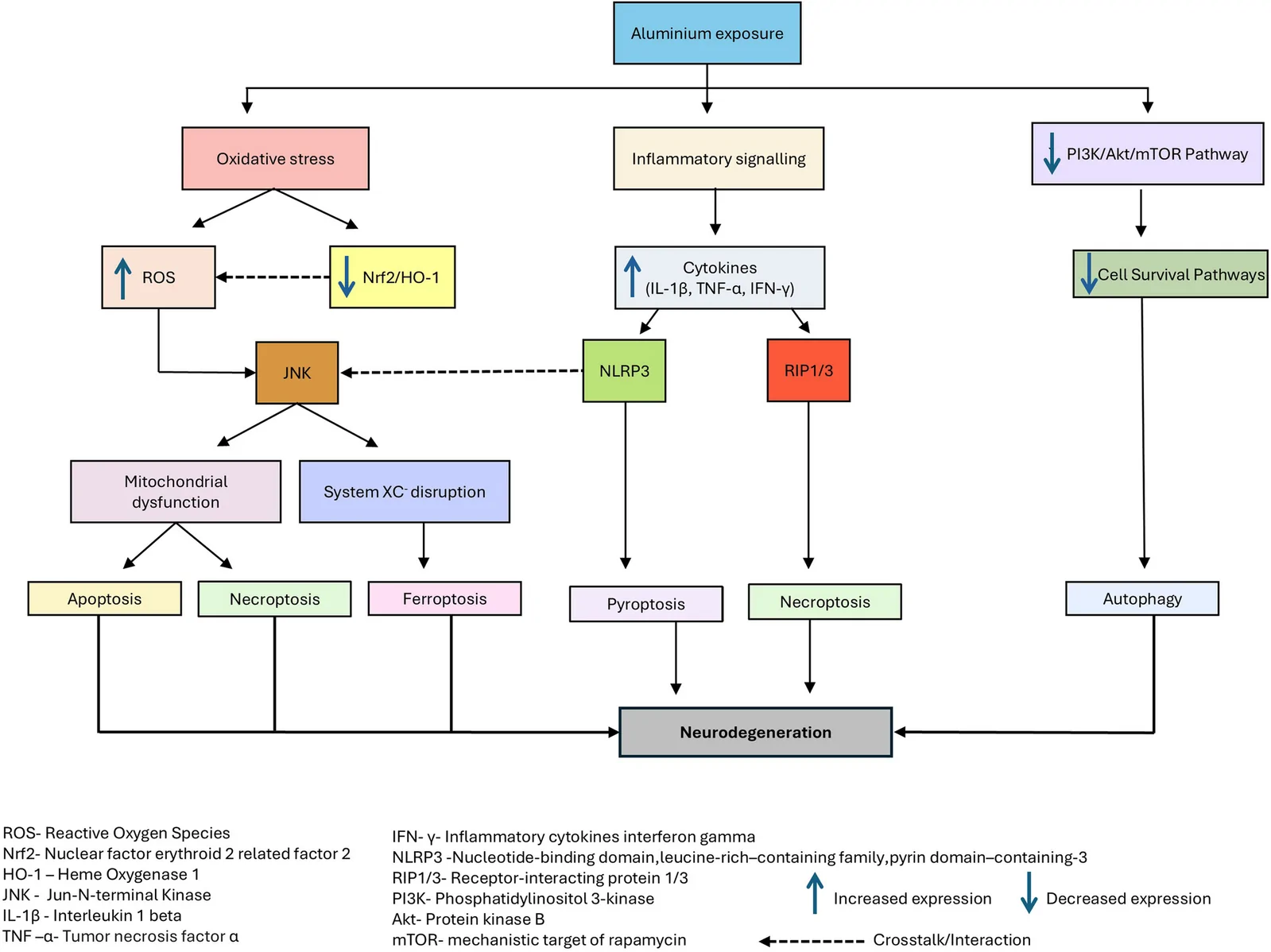
Overview of the interconnected molecular mechanisms of aluminium-induced neurotoxicity Aluminium exposure triggers oxidative stress, inflammatory signalling, and PI3K/Akt/mTOR pathway dysregulation, leading to apoptosis, ferroptosis, pyroptosis, necroptosis, and altered autophagy. Crosstalk between oxidative stress and inflammatory pathways further amplifies neuronal damage, ultimately contributing to neurodegeneration

Inflammatory signalling provides another important link between these pathways. Aluminium exposure elevates the expression of cytokines such as IL-1β, TNF-α, and IFN-γ, which activate apoptosis, necroptosis, and pyroptosis through the JNK, RIP1/RIP3/MLKL, and NLRP3 inflammasome signalling pathways, respectively (Zhang et al. 2020; Hu et al. 2020; Hao et al. 2021, 2023; Zhu et al. 2023). The JNK pathway may represent a key point of convergence, as it contributes to both apoptotic and ferroptotic cell death (Zhang et al. 2020, 2021). Furthermore, inhibition of the PI3K/Akt/mTOR pathway reduces neuronal survival signalling and may facilitate apoptosis and dysregulation of autophagy (Shang et al. 2020; Li et al. 2020). Collectively, these findings indicate that oxidative stress, neuroinflammation, mitochondrial dysfunction, and impaired neuroprotective signalling act together to drive aluminium induced neuronal loss (Fig. 6).

## Conclusion

Exposure to elevated levels of aluminium through various environmental sources can lead to neurodegeneration through neuronal death. The studies reviewed reveal that aluminium, through a variety of mechanisms, can induce various forms of neuronal death. These mechanisms include oxidative stress, mitochondrial dysfunction, neuroinflammation, activation of death signalling and suppression of neuroprotective pathways. These pathways can also be synergistic, establishing a vicious cycle of oxidative stress, inflammation and neuronal injury. Although the precise role of aluminium in neurodegenerative diseases remains unclear, the evidence suggests that aluminium exposure can significantly disrupt cellular homeostasis and promote neurodegeneration. Taken together, these findings imply that aluminium neurotoxicity is the result of a combination of the simultaneous activation of several interconnected cell death pathways and the inhibition of protective pathways. Crosstalk between these mechanisms has the potential to reveal new biomarkers and therapeutic targets for aluminium-associated neurodegeneration, and future research exploring these mechanisms could further improve early diagnosis, prevention and treatment.

## Data Availability

No datasets were generated or analysed during the current study.

## Declarations

### Generative AI and AI-assisted technologies in the writing process

No AI tool was used during the preparation of this paper.
